# Electronic Detection of Functional Cellular Immunity Using Enzymatic Metallization

**DOI:** 10.1021/acsomega.5c11489

**Published:** 2026-02-06

**Authors:** Yuvraj Rallapalli, Josiah Rudge, Madeline Hoyle, Rebecca Corral, Advaith Nair, Mallika Senthil, Caitlin Costello, Aniruddh Sarkar

**Affiliations:** Wallace H. Coulter Department of Biomedical Engineering, 1372 Georgia Institute of Technology, Atlanta, Georgia 30332, United States

## Abstract

Accurate detection of immune cell function at the single-cell level is needed for diagnostic and research applications, yet conventional methods such as flow cytometry require complex instrumentation and fluorescent labeling which limits their use in resource-poor settings. To address this limitation, we developed Electronic Phenotyping using Impedance Cytometry (EPIC), a platform that combines antibody-directed enzymatic metallization on the cell surface with multifrequency impedance analysis, in a microscale 3D-printed plastic aperture, to electronically detect surface marker-based metallization and cytokine secretion. Using CD45-targeted metallization, EPIC generated distinct impedance signatures in Jurkat cells and primary human peripheral blood mononuclear cells, with impedance changes correlating to surface metallization. A bispecific capture strategy coupled to the metallization was then used for detection of IFN-γ secretion of cells, with impedance readouts matching flow cytometry. These findings demonstrate the ability of the EPIC system for sensitive electronic immune profiling and support its potential as a scalable, low-cost alternative to fluorescence-based assays for point-of-care cellular immunity based diagnostics.

## Graphical abstract

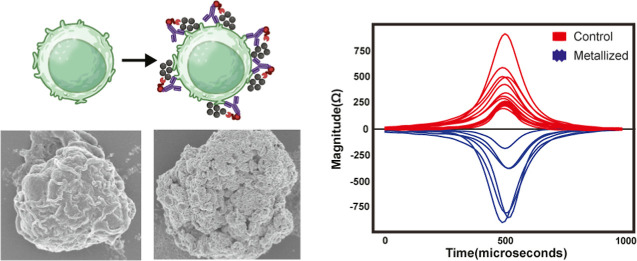

## Introduction

Immune monitoring offers a unique biological lens into how the human body responds to infections, vaccines, and immunotherapies. Traditionally divided into humoral and cellular branches, the immune system responds to infections through antibody production by B cells and antigen-specific activation of T cells and other effector populations, respectively. While pathogen detection-based and antibody-based diagnostics are widely used,^1^ growing evidence suggests that relying solely on pathogen or humoral readouts may overlook critical phases of immune engagement.^2^ Cellular and humoral responses often unfold on different timelines depending on the disease. T cell activation, for instance, can precede detectable antibody production in early stages of infections like tuberculosis (TB) or COVID-19.^3,4^ These dynamics highlight the limitations of relying solely on antibody-based diagnostics and point to a need for more accessible and sensitive tools that can reliably capture cellular immune responses.

Assessing cellular immunity is essential for accurately understanding disease states, especially through markers like antigen-specific cytokine secretion and T cell activation. Common cellular immunity-based diagnostic methods such as Interferon-gamma release assays (IGRAs), ELISpot, and intracellular staining have proven effective in research and clinical laboratories.^5^ IGRA and ELISpot have been adapted and widely used for detection of latent TB infection (LTBI).^6^ ELISpot has also been widely employed in vaccine research and immune monitoring, enabling quantification of antigen-specific T cell responses in clinical trials and immunotherapy studies.^7^ However, they rely on bulky and expensive equipment, including flow cytometers and fluorescence microscopes or require complex sample preparation which limits their use in point-of-care (POC) settings (Table S1).^8,9^ These instruments typically require lasers and photomultipliers to perform immunophenotyping and functional assays with fluorophore-labeled antibodies. While these aspects make the assays highly sensitive and technically robust, the complexity makes it challenging to implement cellular immunity-based diagnostics in low-resource environments, where diseases like TB are often endemic.^10,11^ To bridge this gap, recent research has explored microfluidic chips and electronic detection-based platforms that allow miniaturized detection of cellular immune markers reducing reliance on optical systems and increasing compatibility with POC formats for cellular based diagnostics.^12–22^ Probe-directed enzymatic metallization assays have enabled inexpensive yet sensitive optical and electronic POC antibody profiling in the case of humoral immunity (Table S2).^23–28^ While enzymatic metallization on microparticles has been established, the use of enzymatic metallization for cellular immunity has remained unexplored. In this approach, enzyme-conjugated antibodies can selectively bind to surface markers on immune cells, catalyzing the localized reduction of a soluble metal precursor to deposit conductive material directly at the site of recognition i.e. the cell surface itself, thereby potentially enabling electronic detection of cellular phenotypes.

Here, we introduce an inexpensive microfluidic platform for in-flow, single-cell electronic immunophenotyping, built on antibody-directed enzymatic metallization on cell surfaces (Figure 1A). This approach enables selective surface metallization of immune cells in suspension, which can then be electronically detected through impedance measurements as they pass through the device. The impedance measurement scheme uses multifrequency impedance cytometry to capture changes due to enzymatic metallization directly on cell surfaces.^29–31^ While previous efforts have explored surface-bound cytokine capture,^32–34^ our integration of metallization creates a unique electronic readout that bypasses traditional fluorescence-based methods. In this work, we reliably distinguish metallized cells from control cells based on changes in impedance, quantify the fraction of metallized cells within mixed populations, and demonstrate successful metallization of both a model cell line and primary human immune cells. Furthermore, by capturing secreted cytokines at the cell membrane and inducing localized metallization, we enable electronic detection of cytokine-secreting cells, allowing functional profiling of immune activation without reliance on optical methods. This strategy lays the groundwork for scalable phenotyping of both surface expression and cytokine secretion advancing electronic detection as a viable alternative for cellular immune monitoring. We term this **E**lectronic **P**henotyping using **I**mpedance **C**ytometry or **EPIC**.

**Figure 1:**
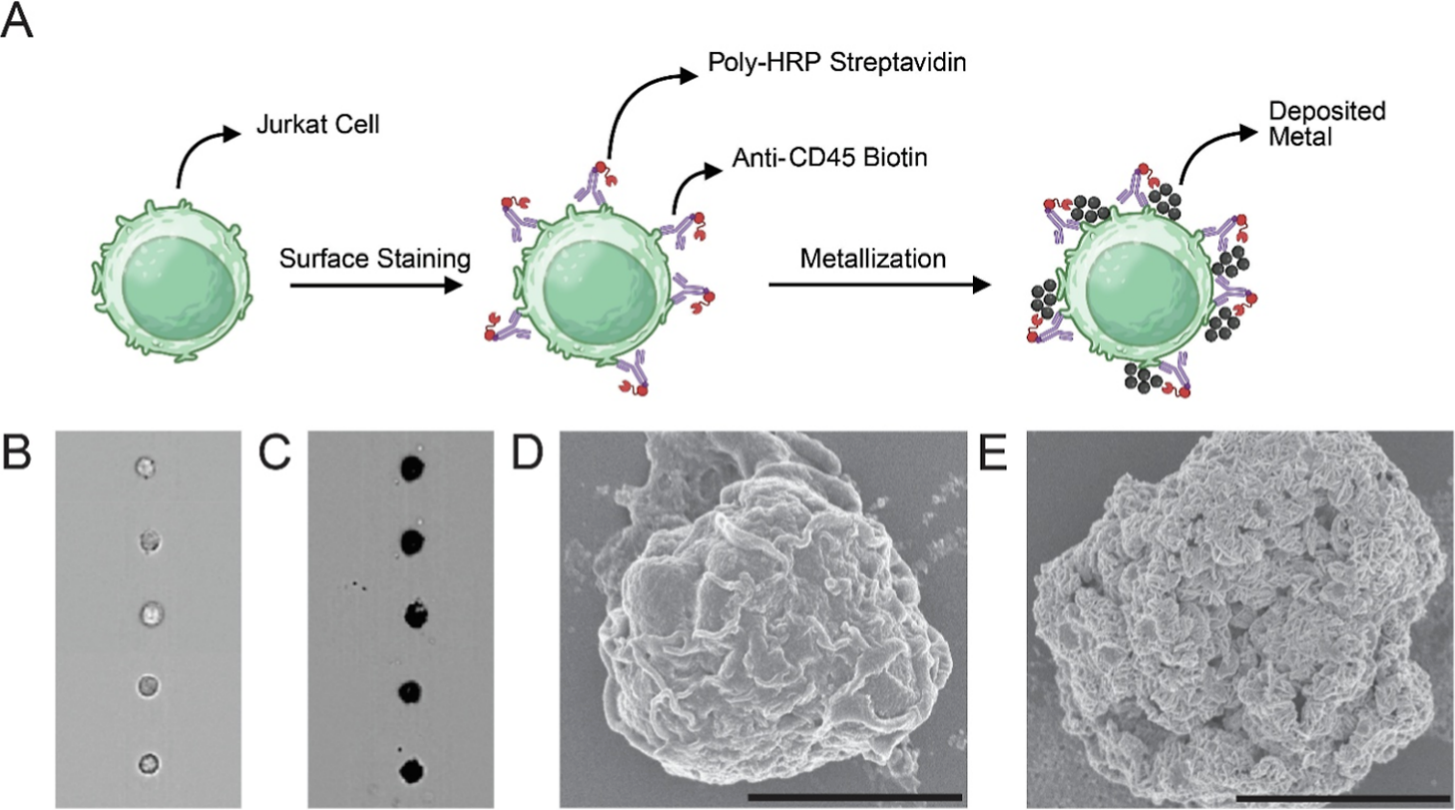
Antibody-directed enzymatic metallization of Jurkat cells. (A) Schematic of workflow illustrating surface labeling of Jurkat cells using anti-CD45 biotin, Poly-HRP streptavidin, and enzymatic silver deposition. (B,C) Imaging cytometry of (B) control and (C) metallized Jurkat cells, demonstrating enhanced contrast following metallization. (D,E) Scanning electron micrograph (SEM) images of (D) control and (E) metallized cells; metallized surfaces exhibit rosette-like nanostructures characteristic of enzymatic silver.

## Results and Discussion

### Enzymatic Metallization of Cells

We first tested probe-directed enzymatic silver deposition on cell surfaces by developing a model assay with Jurkat cells by targeting the abundantly expressed CD45 surface marker (∼200,000 molecules per cell).^35^ In this assay, the cells were incubated with an anti-CD45 biotin probe followed by Poly-HRP streptavidin and enzymatic metallization reagents (Figure 1A). Imaging cytometry revealed high visual contrast between metallized and nonmetallized cells. The morphology of deposited silver metallization was observed using scanning electron microscopy (SEM) as well. We observed the presence of distinct “rosette” shaped silver nanostructures on the surface of cells which is known to be characteristic of enzymatic metallization (Figure 1B–E).^23–26,29^ Figure 1D shows a control cell where no surface metallization was observed. Thus, the deposition of silver was found to be dependent on the presence of HRP on the cell, with minimal background deposition, ensuring its use for a reliable readout for detecting the presence of a surface marker or intracellular protein. Compared to conventional fluorescence-based methods, enzymatic metallization thus potentially offers enhanced signal amplification and higher-resolution surface labeling using brightfield microscopy-based devices while also enabling compatibility with electrical detection workflows. Furthermore, the presence of these distinctive nanostructures on the cell surface could result in a unique electronic signature, which we study next utilizing a microfluidic impedance measurement system.

### Microfluidic Impedance Cytometry of Metallized Cells

To measure the electronic signature of these metallized cells, we utilized an inexpensive microfluidic system where cells pass through a narrow 3D-printed plastic biconical constriction aperture (Figure 2, see Methods for details of fabrication). The constriction in the aperture localizes the electric field in it to a small sensing region allowing for single-cell resolution with no cross talk from other cells close by (Figure 2A). The 3D-printed aperture was visualized using SEM imaging (Figure 2B). Specifically, a 25 μm diameter aperture was positioned between two plated through-hole electrodes on printed circuit boards (PCBs), assembled with a plastic reservoir for loading cells and flow tubing connections (Figure S1). The aperture impedance was measured, via the PCB electrodes, using a lock-in amplifier at six different frequencies ranging from 45 kHz to 20 MHz simultaneously. Cells were loaded into a reservoir and pulled through the aperture at a flow rate of 5 μL/min using a syringe pump. The change in impedance was measured relative to the baseline impedance of the aperture. As a cell moves through the aperture, impedance magnitude at the measurement frequency of 2 MHz was found to increase, peaking as the cell passes the narrowest point before dropping again as the cell exits this region. In contrast, for a metallized cell, impedance magnitude decreased instead (Figure 2C) as the cell passed through the aperture. This difference in impedance magnitude implies that the metallized cells are more conductive than the surrounding fluid (at 2 MHz), and nonmetallized cells are less conductive. The impedance change is symmetrical in time due to the symmetric biconical nature of the aperture.

**Figure 2:**
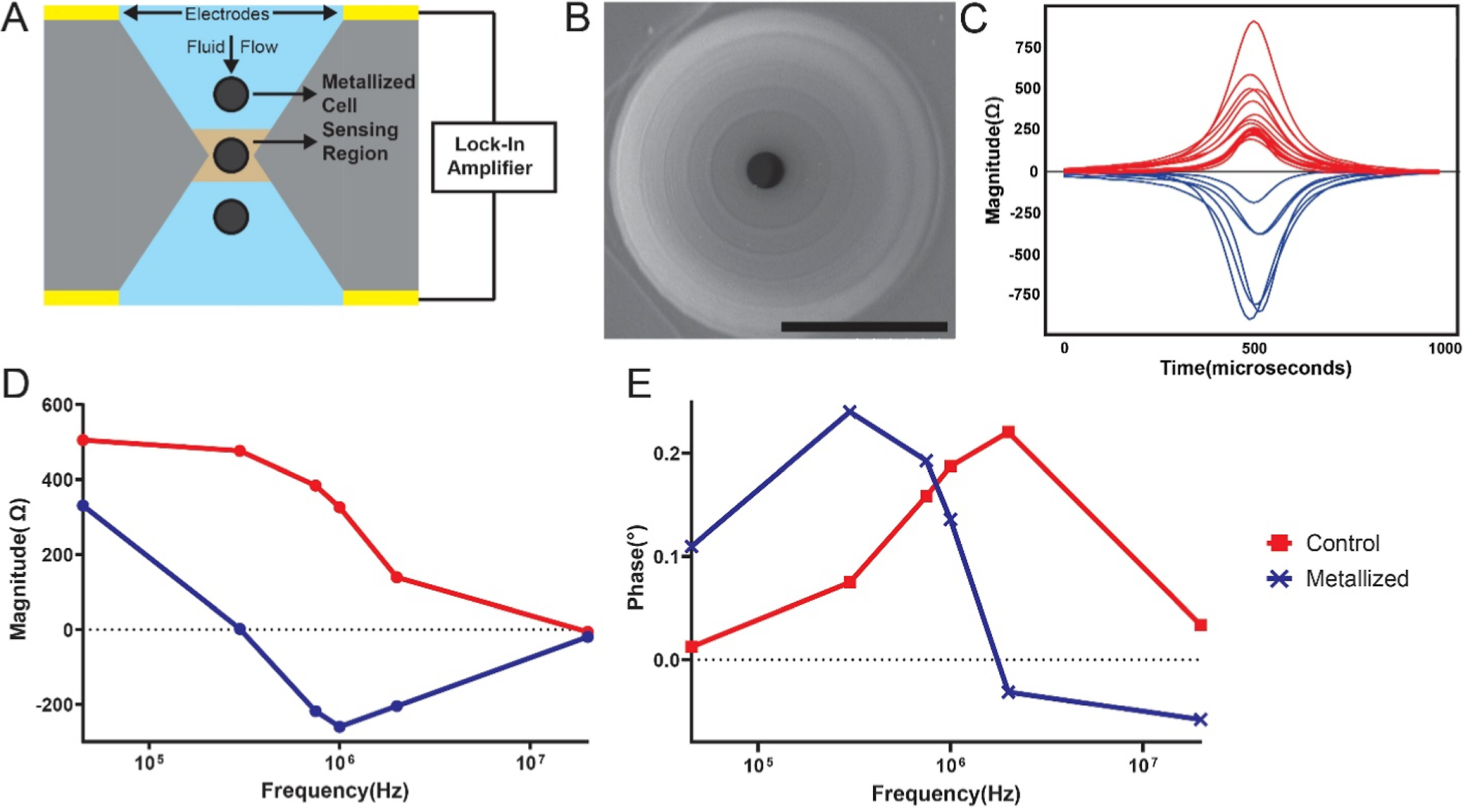
Microfluidic impedance cytometry of metallized cells. (A) Schematic of the biconical constriction aperture with through-hole electrodes on either side. Cells are pulled through this constriction, and impedance is measured in the sensing region using a lock-in amplifier (B) SEM image of the 3D-printed aperture used for single-cell impedance measurements. Scale bar: 100 μm. (C) Representative impedance magnitude traces at a measurement frequency of 2 MHz for control and metallized cells as they flow through the aperture. (D) Mean impedance magnitude spectra for control and metallized cells; metallized cells exhibit reduced magnitude at higher frequencies. (E) Mean phase spectra showing increased phase at low frequencies and reduced phase at high frequencies for metallized cells. *n* = 1000 for both conditions.

The impedance of each cell passing through the sensing region is measured at all six frequencies simultaneously using the lock-in amplifier, providing multifrequency single-cell impedance magnitude and phase spectra, which are captured rapidly for each single cell (∼1 ms per cell) providing high single-cell throughput (>1000 cells per minute). This is visualized with the help of Bode plots, which showcase the median response of the cells across the six frequencies. Figure 2D,E shows the change in impedance of metallized cells and nonmetallized cells. At low frequencies (45 kHz), metallized cells showed minimal change in magnitude relative to nonmetallized cells. In contrast, at higher frequencies (>300 kHz), metallized cells exhibited a pronounced negative change in magnitude, resulting in a distinct impedance spectral profile compared to nonmetallized cells (Figure 2D). The corresponding phase spectrum of metallized cells shows an increase at low frequencies and a substantial reduction at higher frequencies, with a crossover occurring around 1–2 MHz (Figure 2E). Although changes in magnitude were minimal at 20 MHz, a pronounced difference in phase was observed between metallized and nonmetallized cells.

While a priori the metallized cell surface maybe expected to behave as a fully connected conductive shell, producing a higher conductivity and hence negative shifts in impedance magnitude even at low frequencies, the measured data did not support this hypothesis. At 45 kHz, the impedance magnitude remains largely unchanged between metallized and control cells. Although the phase spectrum shows some deviation, the overall response suggests limited conductive behavior at this frequency. These findings indicate that the metallization may not form a continuous conductive layer but instead may create a discontinuous mesh interspersed with fluid interfaces. At higher frequencies, the influence of this mesh becomes more pronounced, resulting in reductions in both magnitude and phase that are consistent with capacitive effects arising from the metal-fluid interfaces within the mesh.^29^ This frequency-dependent behavior thus yields a distinct spectral profile that allows clear differentiation between metallized and nonmetallized cells.

### Electronic Identification of Selectively Metallized Cell Mixtures

Having tested metallized and nonmetallized cells separately, we wanted to next evaluate the sensitivity of the EPIC scheme in detecting small subpopulations of metallized cells within a nonmetallized cell population background in a mixture of both kinds of cells (Figure 3). A key question we sought to answer is, whether unlabeled cells could be nonspecifically metallized by neighboring labeled cells, when they are in the same cell suspension. We designed an experiment where anti-CD45 labeled cells were spiked within unlabeled control cells at varying concentrations. (e.g., the 40% group consists of 40% labeled and 60% unlabeled cells (Figure S2)). These mixed cell suspensions were then metallized by addition of the enzymatic metallization reagents after the mixtures were formed. We observed the median change in magnitude of the mixed cell suspension, after metallization, reduces as the labeled cell concentration increases at high frequencies, with small changes at low frequencies. This can be attributed to the number of metallized cells increasing with labeled cell concentration (Figure 3A). We see a similar change in phase as well, with the phase increasing with higher labeled cell concentrations at low frequencies and reducing at higher frequencies (Figure 3B).

**Figure 3:**
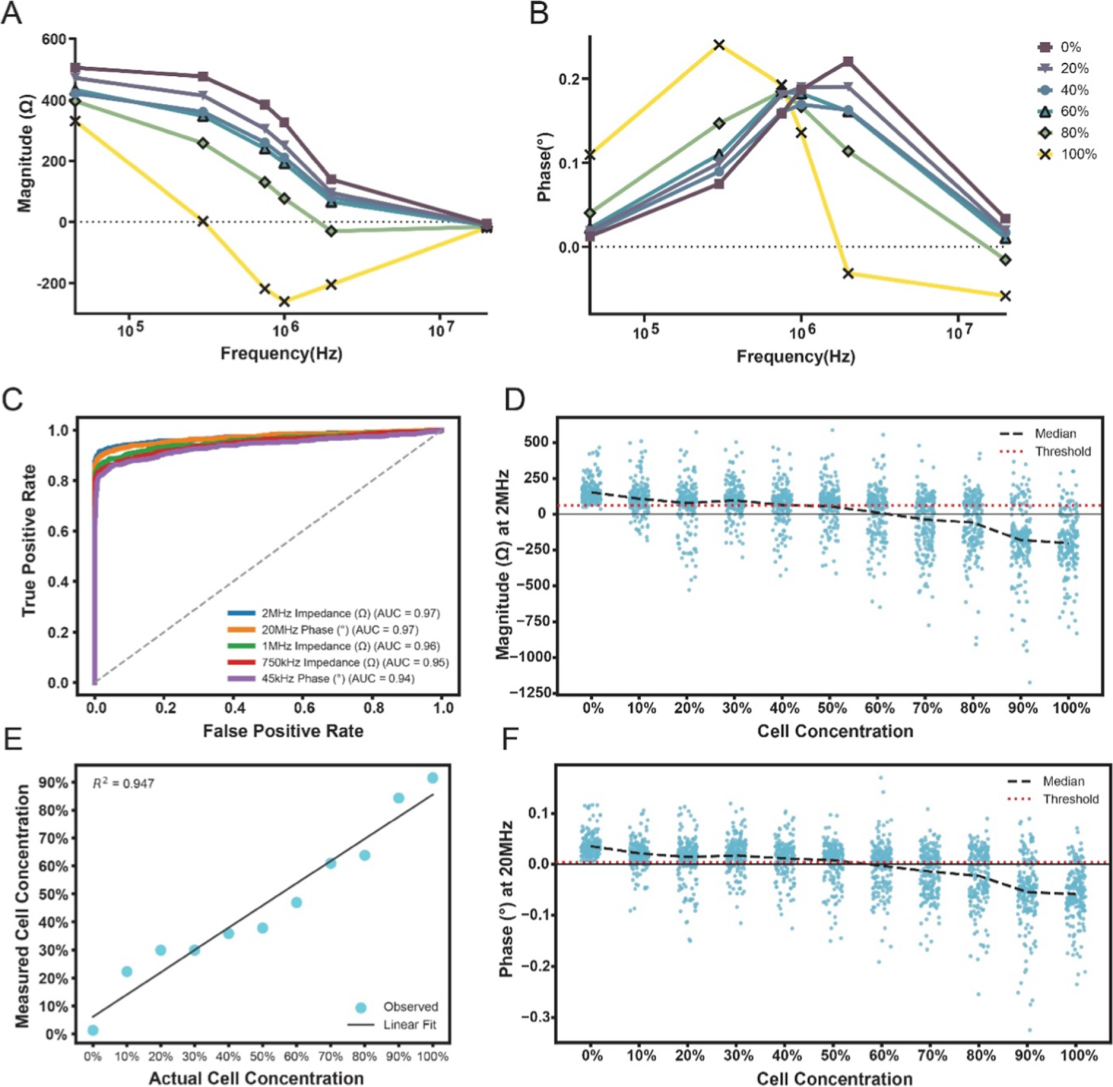
(A) Median impedance magnitude spectra for mixtures of cells containing defined fractions of labeled and unlabeled Jurkat cells (*n* = 1000 cells per condition). Magnitude decreases at higher frequencies as the concentration of labeled cells increases. (B) Mean phase spectra for the same mixtures (*n* = 1000 cells per condition); phase increases at low frequencies and decreases above 1 MHz with increasing labeled cell concentration. (C) Receiver operating characteristic (ROC) curves distinguishing 0% (all unlabeled) from 100% (all labeled) samples; the five top features by AUC are shown. (D) Impedance magnitude at 2 MHz versus labeled-cell concentrations, with mean trendline and an optimal threshold derived from a binary decision tree classifier. For visualization, a subsample is shown (*n* = 200 events per condition). (E) Measured metallization versus actual labeled cell concentration using the 2 MHz magnitude threshold; linear fit *R* ^2^ = 0.947. (F) Phase at 20 MHz versus labeled cell concentration, with mean trendline and the corresponding threshold.

To evaluate the best metric for distinguishing between control and metallized cells, we studied the receiver operator characteristic (ROC) curve of each of the 12 measured impedance features to distinguish between the 0% labeled and 100% labeled samples. The best five discriminative features are shown in Figure 3C, the best of which are impedance magnitude at 2 MHz and phase at 20 MHz both with an AUC of 0.97. To find the optimal threshold for separation, a binary decision tree classifier was used for each individual feature.^36^ Figure 3D shows the change in magnitude at 2 MHz as labeled cell concentration increases. The median of the measured single cells (*n* = 1000 each) and optimal threshold as calculated are overlaid as well. The median impedance magnitude reduces as labeled cell concentration increases. However, the spread of each sample also increases. This is possibly due to the heterogeneity of the cell metallization compounded by the inherent heterogeneity of cell sizes as well.

Despite this heterogeneity, ability to predict the metallized cell concentration of each population was demonstrated, with cells above the threshold considered as nonmetallized and cells below the threshold as metallized cells. As shown in Figure 3E a linear relationship was observed between actual cell concentration and measured metallized cell concentration with an *R* ^2^ value of 0.947 showing a good fit and the ability to accurately measure the metallized subpopulations of cell mixtures. Cell concentrations as low as 0.5% were detectable, showcasing the ability of EPIC for rare event detection as well (Figure S3). Similarly, Figure 3F shows the change in phase at 20 MHz as the cell concentration increases, notably the optimal threshold for this feature was ∼0, with control cells having a positive change in phase and metallized cells having a negative change in phase. Overall, these linear changes as metallized cell concentration increases indicate that there is minimal cross reactivity between cells, with metallization on cell surface being due to the presence of bound poly-HRP, and not from neighboring cells. Notably, when the impedance spectra of cells above and below the 2 MHz threshold were plotted, all the samples showed a similar trend irrespective of the cell concentration, further confirming the lack of cross reactivity, and homogeneous metallization (Figure S4). Thus, we show the ability of the EPIC system to identify cellular subpopulations, with a clear threshold differentiating between metallized and nonmetallized cells.

### PBMC Immunophenotyping via Surface Metallization

Building upon the prior validation of EPIC in Jurkat cells, we next extended the platform to primary human peripheral blood mononuclear cells (PBMCs). We also intended, in these experiments, to determine the suitability of CD45 as an anchoring site for subsequent surface-captured cell-secreted cytokine detection. CD45 was selected based on its broad and elevated expression across leukocyte subsets, hypothesized to facilitate sufficient enzymatic metallization for inducing impedance changes (Figure 4A). Consistent with the above findings from Jurkat cells, anti-CD45-labeled PBMCs, after metallization, exhibited minimal change in impedance magnitude at low frequencies and a marked decrease at higher frequencies (Figure 4B). Likewise, the phase response demonstrated an increase at low frequencies and a decrease at high frequencies (Figure 4C). Similar responses were obtained with isolated T cells, confirming that anti-CD45 driven metallization retains a consistent electrical signature across cell types including primary calls as well (Figure 4D,E). The magnitude of change was found to be more pronounced in PBMCs and T cells than in Jurkat cells, likely due to an increased metallization layer-to-cell size ratio, which can enhance the capacitive contribution of the metallization layer at the cell–liquid interface.

**Figure 4:**
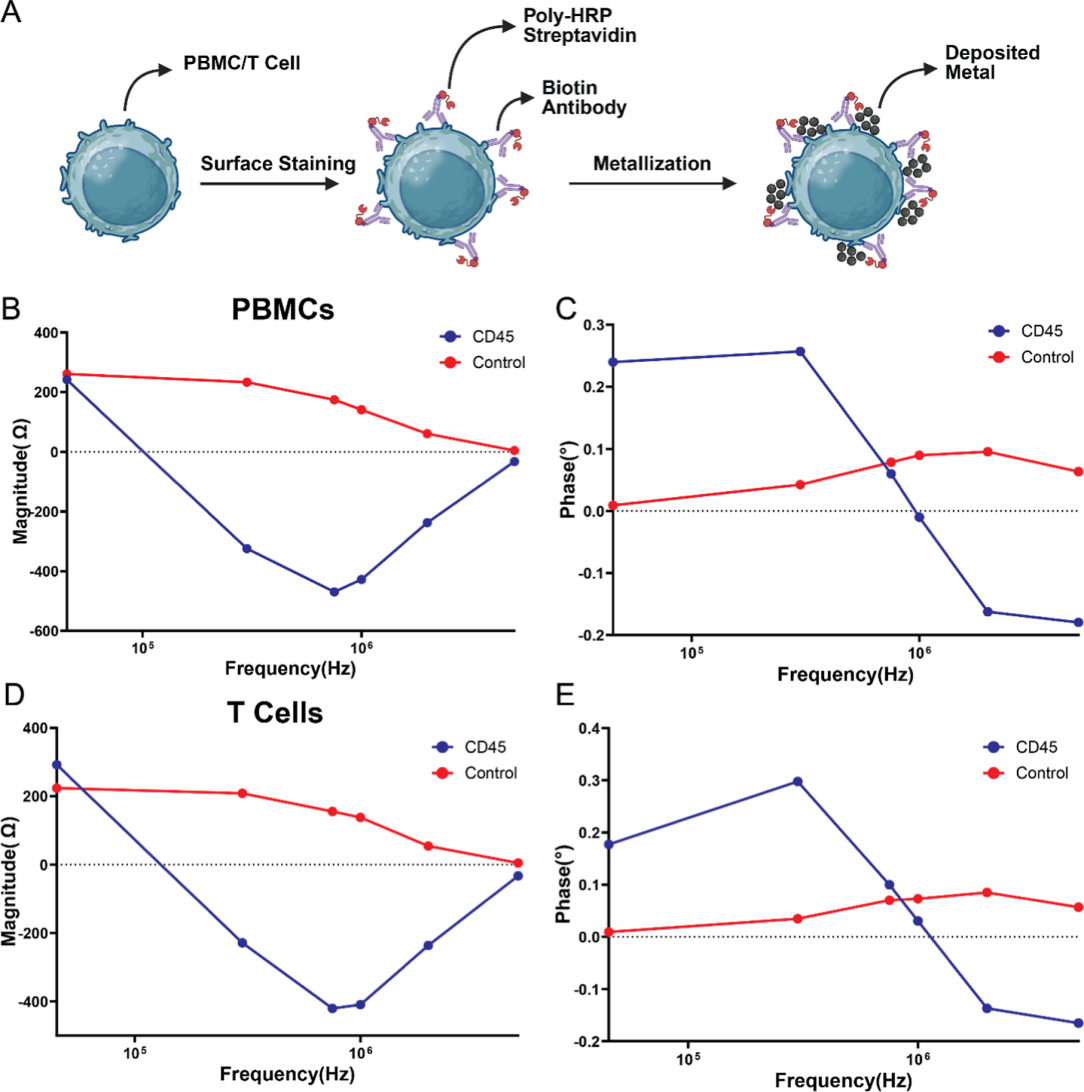
Impedance cytometry of anti-CD45-labeled primary human PBMCs and T cells. (A) PBMCs and isolated T cells were stained with anti-CD45 antibody followed by enzymatic metallization. (B) Impedance magnitude spectra for anti-CD45-labeled PBMCs showed negative change in magnitude at higher frequencies compared to unstained controls. (C) Phase spectra revealed increased phase at low frequencies and reduced phase at high frequencies, consistent with metallization-dependent interfacial changes. (D) Impedance magnitude spectra for anti-CD45-labeled T cells exhibited similar reductions at high frequencies. (E) Phase spectra for T cells showed the same metallization signature observed in PBMCs. *n* = 200 for all conditions.

To further characterize the responsiveness of EPIC to surface-bound metallization, we additionally tested other surface markers of cells present in PBMCs: CD3, CD14, and CD19 for T cells, monocytes, and B cells, respectively. These markers were selected to test whether EPIC could serve as a viable tool for direct surface marker–based electrical immunophenotyping. However, in the current assay and detection scheme, none of these probes yielded appreciable shifts in impedance magnitude or phase relative to control samples, either in mixed PBMC populations or in isolated T cell subsets (Figures S5 and S6). This observation is likely attributable to the comparatively higher surface expression of CD45 on PBMCs relative to CD3, CD14, and CD19 ^35,37,38^ which enables more extensive enzymatic metallization and sufficient metal deposition to induce capacitive interactions at the cell–liquid interface. These results suggest that the impedance changes in EPIC are governed not only by marker presence but also by the density of surface expression, which determines the extent of metal-fluid interface formation.

These results suggest that the impedance changes in EPIC are governed not only by surface marker presence but also by the density of surface expression. While CD45 provided a robust signal due to its high expression levels, detection of lower-abundance markers may require additional signal amplification, which is a limitation of our present work. Future iterations of EPIC could integrate Gold Nanoparticles (GNPs) or Biotinyl-Tyramide (BT) signal amplification to enhance metallization kinetics, thereby enabling the resolution of markers such as CD3, CD14, and CD19 that exhibit lower surface densities. Additionally, the ability of the system to capture cell size via baseline impedance presents an opportunity to multiplex size data with CD45 metallization. This dual-parameter analysis could enable the electronic discrimination of WBC subpopulations, facilitating the generation of rapid differential counts without additional labeling.

### Detection of IFN-γ Secretion via Surface Capture and Metallization

Finally we evaluated the performance of EPIC in detecting cell-secreted cytokines, specifically, interferon gamma (IFN-γ). PBMCs were stimulated overnight with PMA ionomycin stimulation cocktail to induce IFN-γ production. A bispecific capture antibody was bound to the cell surface, followed by a 3 h incubation to capture secreted IFN-γ (Figure 5A). This bispecific approach was adapted to enable metallization by incorporating an APC-conjugated detection antibody, followed by anti-APC biotin and streptavidin-polyHRP. This indirect labeling strategy allowed us to amplify the signal and selectively metallize cells with surface-bound IFN-γ while allowing comparison with fluorescence-based detection using flow cytometry as well. Preliminary testing was performed using Jurkat cells dosed with recombinant IFN-G at varying concentrations. Detection limits using impedance magnitude at 2 MHz were comparable to flow cytometry using the same sample, where IFN-G concentrations as low as 25 ng/mL per 10^6^ cells was detectable (Figure S7).

**Figure 5:**
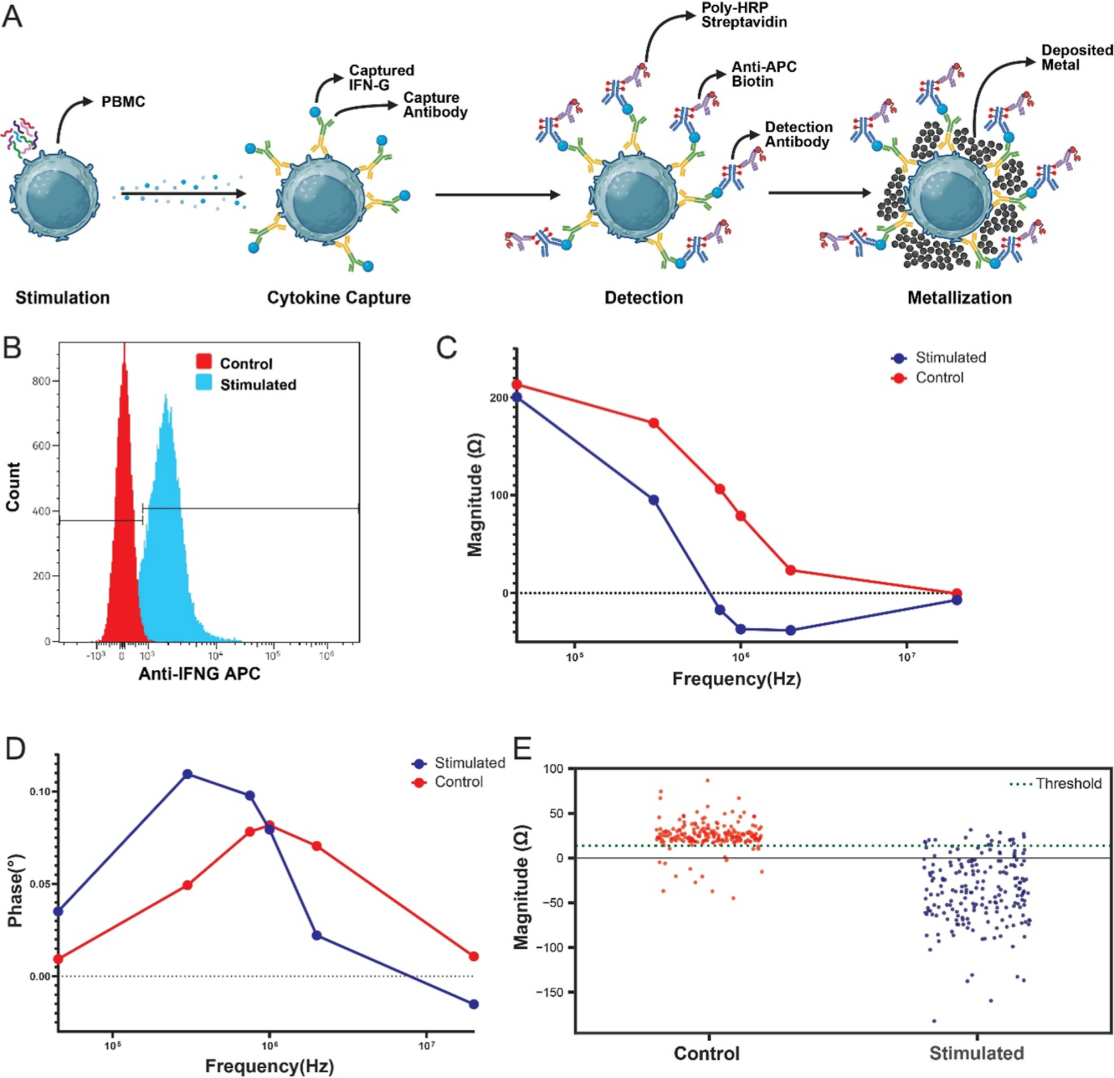
Functional detection of cell-secreted IFN-γ via surface capture and enzymatic metallization (A) Stimulated PBMCs were functionalized with a surface-anchored capture antibody, followed by a cell stimulation and incubation to capture IFN-γ, and labeled with an APC-conjugated detection antibody to enable silver deposition. (B) Flow cytometry confirmed increased surface IFN-γ expression in stimulated cells, with 89% of cells having captured IFN-γ. (C) Impedance magnitude spectra showed negative change in impedance magnitude at high frequencies in stimulated cells compared to controls. (D) Phase spectra revealed increased phase at low frequencies and decreased phase at high frequencies, consistent with metallization. (E) Classification using a binary decision tree threshold at 2 MHz identified that 91% of stimulated cells were metallized, in agreement with flow cytometry estimates. *n* = 200 for both conditions.

Following PBMC stimulation, flow cytometry confirmed successful cytokine capture, with 89% of cells exhibiting surface-bound IFN-γ (Figure 5B). To maximize capture efficiency, here, cells were incubated in a low-volume suspension, increasing the effective concentration of secreted cytokine. This approach was deliberately chosen to prioritize detection sensitivity over single-cell specificity here, reflecting the eventual diagnostic context where detecting as many secreting cells as possible is critical. However, we note here that the elevated concentration of IFN-γ in the medium, in this experiment, may lead to cross-binding of secreted cytokine across nearby cells, resulting in higher sensitivity of IFN-γ detection, with lower single cell specificity.

Following metallization, as earlier, impedance measurements showed minimal changes in magnitude at low frequencies relative to controls, but a significant reduction at higher frequencies, consistent with prior metallization profiles (Figure 5C). Phase spectra similarly showed elevated phase change at low frequencies and reduced phase at high frequencies (Figure 5D). These changes were less pronounced than in earlier experiments, likely due to the added complexity of the antibody capture and detection stack, which may have led to lower or less uniform metallization.

At a measurement frequency of 2 MHz, impedance magnitude provided strong separation between stimulated and control populations. Using the previously defined decision tree threshold, 91% of stimulated cells were classified as metallized, compared to 11% in the control group (Figure 5E). Phase measurements at 20 MHz yielded similar results, with 92% of stimulated cells classified as metallized, though 28% of control cells were also classified as metallized (Figure S8). These results closely match flow cytometry data and confirm the ability of EPIC to sensitively detect IFN-γ secretion. These findings demonstrate that EPIC can electronically detect cell cytokine secretion, indicating its potential as a functional immune profiling and diagnostic tool beyond traditional fluorescence-based methods.

While the current study utilized commercially available APC-conjugated antibodies for validation, the EPIC platform does not inherently require any fluorescent reporters. A completely fluorescent-labeled antibody free workflow can be achieved by substituting the commercial detection antibody with a directly biotinylated anti-IFN-G antibody. This would allow the cytokine–capture complex to metallize the cell surface via biotin–streptavidin interactions, thereby streamlining the assay and reducing reagent complexity.

To evaluate the potential of EPIC, we benchmarked the projected performance of EPIC against established clinical standards for functional immune monitoring: Intracellular Cytokine Staining (ICS) via flow cytometry and Clinical ELISpot (e.g., T-SPOT.TB).^39,40^ As detailed in Table S1, while flow cytometry remains the standard for high-content multiparametric analysis, EPIC may offer a distinct advantage in accessibility with an estimated instrumentation cost of under USD5,000 (with custom electronics), representing a significant potential reduction compared to clinical flow cytometers (>USD100,000) or automated ELISpot readers (USD25,000–USD75,000).^41^ Furthermore, the streamlined workflow of EPIC enabled by microfluidic integration and rapid enzymatic metallization could potentially reduce the total sample-to-answer time to approximately 6.5 h. This suggests the utility of EPIC as a rapid alternative to the 18–24 h turnaround times necessitated by the overnight incubation protocols typical of clinical ICS and ELISpot assays.^42^

## Conclusion

In this study, we introduced **E**lectronic **P**henotyping using **I**mpedance **C**ytometry or **EPIC** as a platform for electronic detection of immune cell function through antibody-directed enzymatic silver metallization on cell surfaces and multifrequency impedance cytometry in a 3-D printed plastic microaperture. EPIC enables selective surface metallization labeling of immune cells in suspension and generates distinct single-cell electronic signatures that correlate with surface marker expression as well as activation state and functional output such as cytokine secretion. We validated the approach using CD45-targeted metallization on Jurkat cells and primary human PBMCs and isolated T cells, revealing distinct impedance and phase spectral shifts consistent indicative of capacitive behavior of the deposited nanoscopic metallization layer at the cell–liquid interface. These shifts were more pronounced in PBMCs and T cells compared to the cell line which was attributed to increased metallization layer-to-cell size ratios.

We further demonstrated the sensitivity of EPIC in identifying metallized subpopulations within mixed samples, showing a linear correlation between actual and predicted cell concentrations (*R* ^2^ = 0.947) and minimal cross-reactivity, as well as the detection of cell concentrations as low as 0.5%. Additional markers (CD3, CD14, CD19) did not yield significant impedance changes, reinforcing the importance of surface antigen density in signal generation and the need for future work in enhancing the metallization density or electronic detection sensitivity or both. EPIC was also adapted to detect IFN-γ secretion using a bispecific antibody-based surface capture strategy, achieving high classification accuracy for stimulated cells based on measured impedance magnitude and phase features. These results closely matched flow cytometry data and confirmed the ability of EPIC to electronically detect cytokine secretion and measure functional state of PBMCs.

EPIC represents an advancement toward scalable, point-of-care cellular diagnostics. While flow cytometry remains the gold standard for immune profiling, it requires complex optics, fluidics, and instrumentation that limit its accessibility in low-resource settings. In contrast, EPIC relies on electrical readouts that are inherently compatible with miniaturized and portable formats. Although the current implementation utilizes a lock-in amplifier to acquire multifrequency impedance spectra, our results demonstrate that a single frequency specifically 2 MHz for magnitude is sufficient to differentiate metallized from nonmetallized cells with high accuracy. This finding suggests that future systems could be simplified to operate at one or two optimized frequencies, substantially reducing hardware complexity and cost. Such a configuration, combined with higher throughput (>10000 cells/min), would enable deployment of EPIC as a low-cost, portable alternative to flow cytometry for functional immune monitoring in decentralized or resource-limited environments.

## Materials and Methods

### Cell Culture

Jurkat T cells (ATCC, Cat# TIB-152) were cultured in RPMI 1640 medium (Gibco, Cat# 11875–093) supplemented with 10% fetal bovine serum (FBS; Gibco, Cat# 26140–079) and 1% penicillin–streptomycin (Gibco, Cat# 15140–122). Cryopreserved peripheral blood mononuclear cells (PBMCs) and purified T cells were obtained from STEMCELL Technologies (PBMCs: Cat# 70025; T cells: Cat# 70042) and cultured under identical conditions. Cells were maintained at 37 °C in a humidified incubator with 5% CO_2_ and passaged every 2–3 days.

### Aperture Fabrication

The microscale aperture was fabricated via two-photon lithography (Nanoscribe Photonic Professional GT2) based on a Fusion 360 CAD model. Postprinting, the structure was developed in SU-8 developer (10 min), rinsed in isopropanol (10 min), and UV-cured overnight prior to assembly.

### Model Assay for Enzymatic Metallization

One × 10^6^ Jurkat cells were first incubated with 1 μg of biotinylated anti-CD45 antibody (Thermo Fisher, Cat# 13–0459–82) for 30 min at room temperature. Cells were then fixed using Fixation Buffer (Thermo Fisher, Cat# FB001) for 10 min. Following fixation, cells were washed with phosphate-buffered saline (PBS) containing 1% bovine serum albumin (BSA; Sigma-Aldrich, Cat# A2153) and incubated with 2 μg of Poly-HRP streptavidin (Thermo Fisher, Cat# N200) for 30 min. Cells were washed twice and resuspended in 200 mM HEPES buffer. Enzymatic silver metallization was performed using EnzMet reagents A, B, and C (Nanoprobes, Cat# 6010), added sequentially for 4, 4, and 30 min, respectively, at room temperature. Metallization was terminated by washing with PBS.

Imaging Cytometry was performed using Amnis MKII Imaging Cytometer, SEM images were taken using Hitachi S-3700N VP-SEM.

### Immunophenotyping Assay

Cryopreserved PBMCs were thawed rapidly in a 37 °C water bath and resuspended in PBS containing 1% BSA. Cells were incubated with biotinylated antibodies targeting CD3 (Thermo Fisher, Cat# 13–0037–82), CD14 (Thermo Fisher, Cat# 13–0149–82), CD19 (Thermo Fisher, Cat# 13–0149–82), or CD45 for 30 min at room temperature. Following antibody staining, cells were fixed for 10 min. After washing, cells were incubated with 2 μg of Poly-HRP streptavidin for 30 min. Cells were then washed twice and resuspended in 200 mM HEPES buffer. Enzymatic silver metallization was performed using EnzMet reagents A, B, and C, added sequentially for 4, 4, and 30 min, respectively, at room temperature. Metallization was terminated by washing with PBS.

### Cytokine Capture Assay

PBMCs were resuspended in complete RPMI medium and stimulated overnight with PMA/Ionomycin stimulation cocktail (Thermo Fisher, Cat# 00–4970–93) to induce IFN-γ secretion. Cytokine capture was performed using the IFN-γ Secretion Assay – Detection Kit (APC) (Miltenyi Biotec, Cat# 130–090–433), following manufacturer instructions. After a 3 h capture incubation, cells were stained with 2 μg of biotinylated anti-APC antibody for 30 min at room temperature. Cells were then fixed, washed, and incubated with 2 μg of Poly-HRP streptavidin for 30 min. Following two washes, enzymatic metallization was performed using EnzMet reagents A, B, and C, added sequentially for 4, 4, and 30 min, respectively. Metallization was terminated by washing with PBS.

Flow cytometry was performed using the Cytek Aurora 5L Spectral Cytometer (UV–V–B-YG-R), sampled before the addition of the biotinylated antibody.

### Impedance Cytometry

A lock-in amplifier (HF2LI, Zurich Instruments) coupled with a transimpedance amplifier (HF2TA) was used for impedance measurements. The impedance spectra were captured at 6 frequencies (45 kHz, 300 kHz, 750 kHz, 1, 2, and 20 MHz) and sampled at 57.8k samples per second. For waveform identification, the impedance magnitude at 45 kHz is used in a peak finding algorithm with a threshold of 250 Ω. Timestamps of peaks at this frequency are used to directly index the remaining 5-magnitude and 6-phase time-series measurements. The flow rate of the syringe pump was 5 μL/min.

## Supplementary Material



## Notes

Figure S1: Fully assembled aperture. Figure S2: Schematic showing labeled cell mixtures of Jurkats. Figure S3: Impedance magnitude and phase change spectra of CD3, CD14, CD19 and control of PBMCs. Figure S4: Impedance magnitude and phase change spectra of CD3 and control of T Cells. Figure S5: Change in 20 MHz Phase between stimulated and control cells (PDF)

## Abbreviations

PBMC: peripheral blood mononuclear cells
IGRA: interferon-gamma release assays
EPIC: electronic phenotyping using impedance cytometry.
