# Supplementary material for: Electronic Detection of Functional Cellular Immunity Using Enzymatic Metallization
Source: ACS Omega. 2026 Feb 6;11(9):14937–46. doi: 10.1021/acsomega.5c11489 (PMC12980182; doi:10.1021/acsomega.5c11489)
Supplement: Supplementary file 1 [file ao5c11489_si_001.pdf]

Electronic Detection of Functional Cellular Immunity using Enzymatic Metallization

Yuvraj Rallapalli, Josiah Rudge, Madeline Hoyle, Rebecca Corral, Advaith Nair, Mallika Senthil, Caitlin Costello, Aniruddh Sarkar*

Wallace H. Coulter Department of Biomedical Engineering, Georgia Institute of Technology, Atlanta GA 30332

KEYWORDS. Single-cell analysis, Electronic detection, Silver nanoparticles

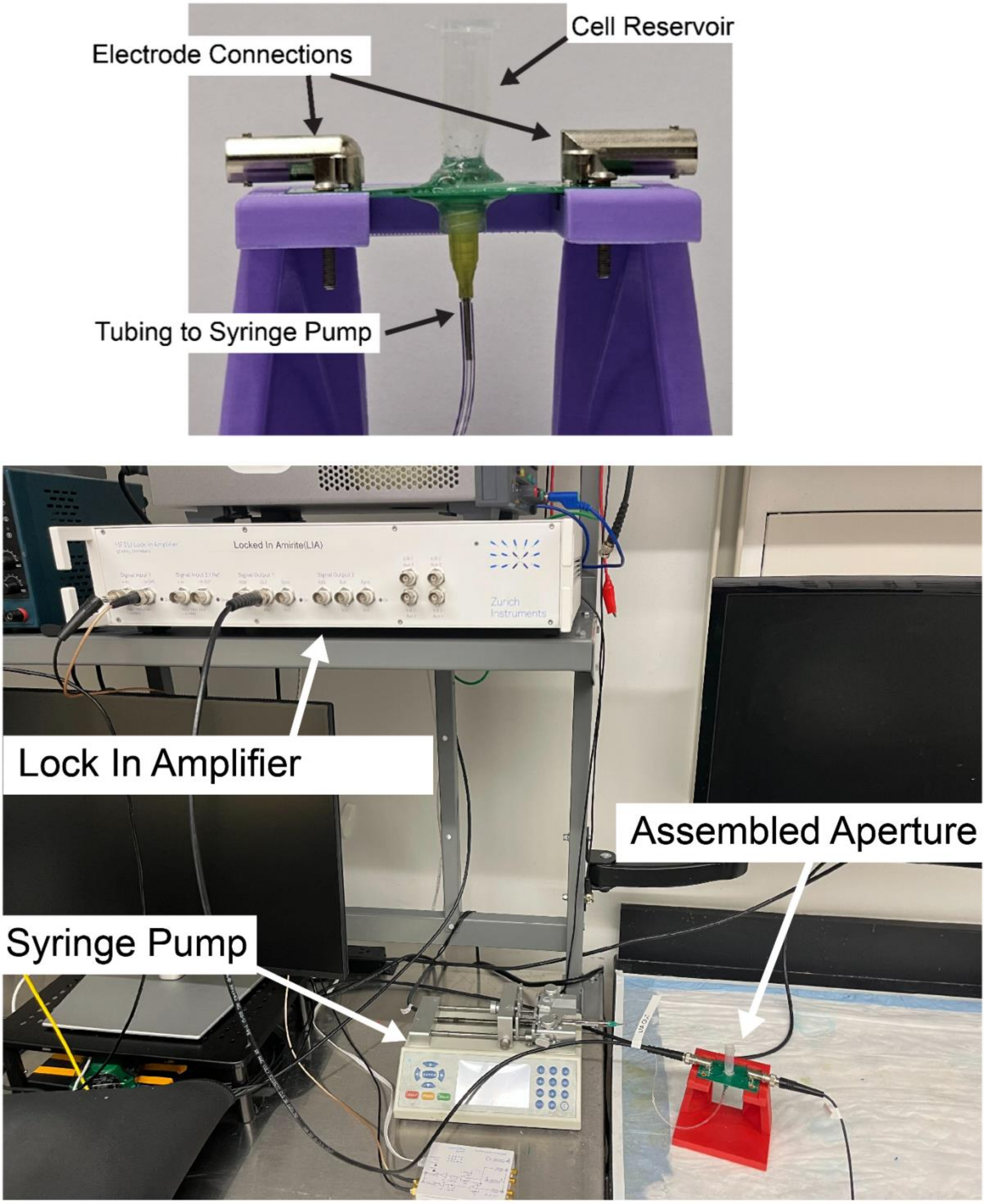

Figure S1: Fully assembled aperture (Top) and experimental setup for impedance detection (Bottom)

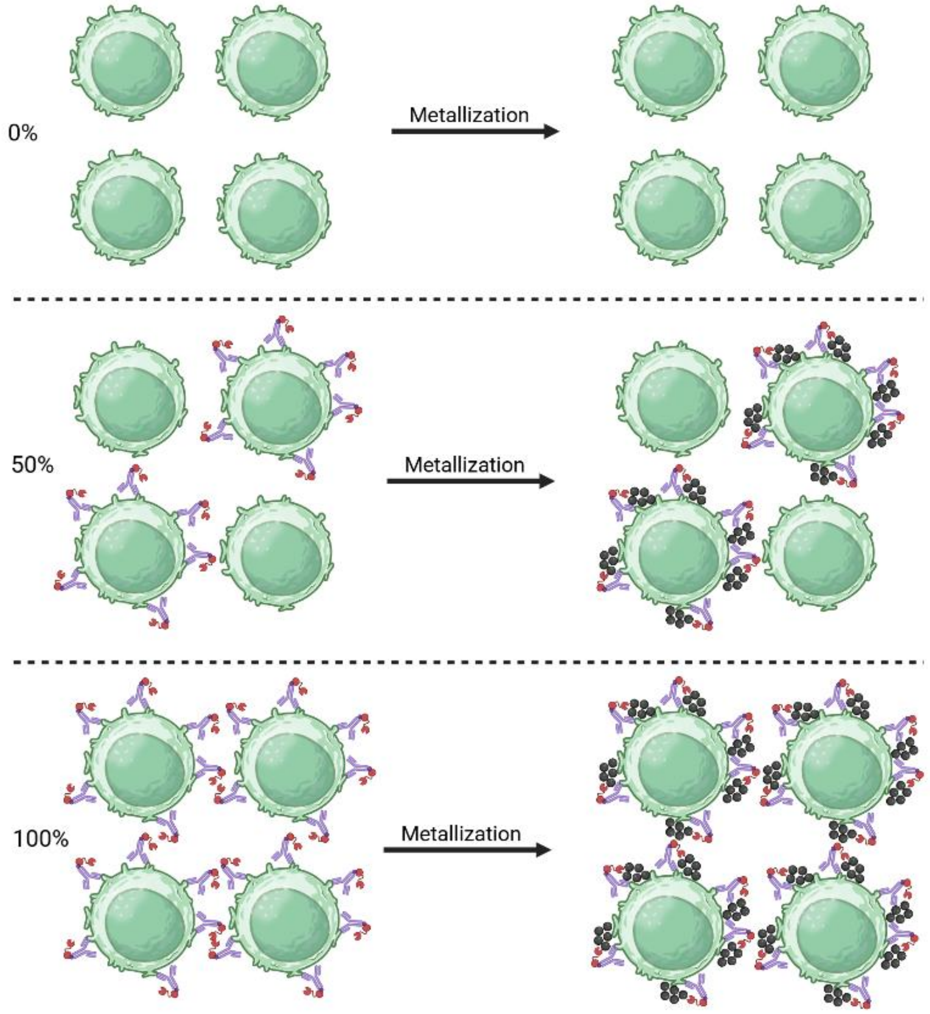

Figure S2: Schematic showing labelled cell mixtures of Jurkats.

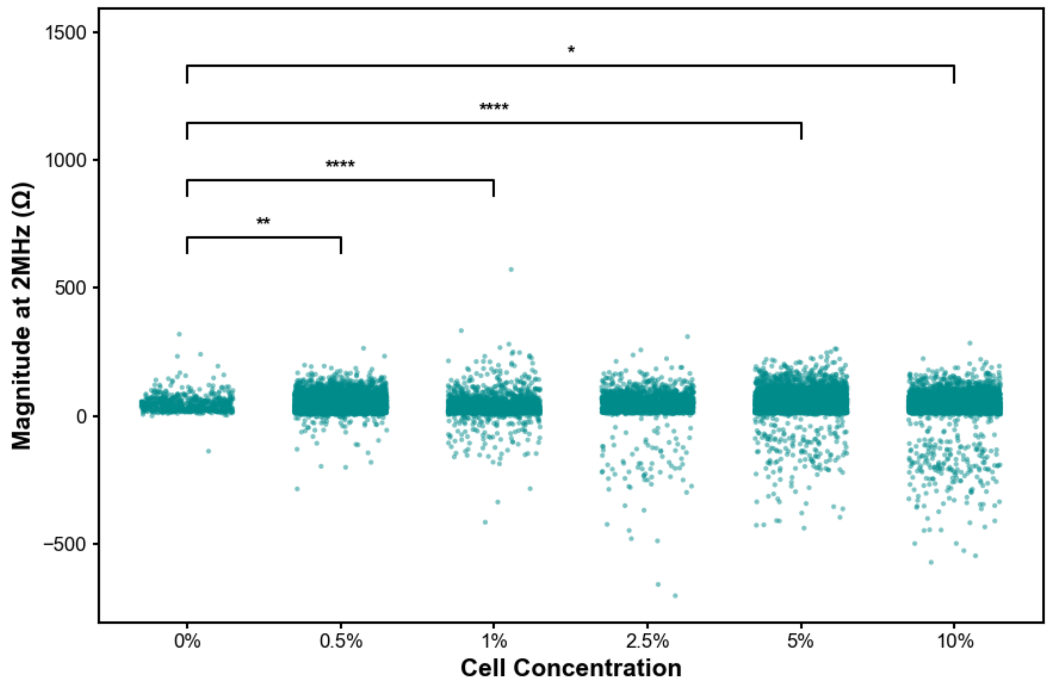

Figure S3: Distribution of cell impedances based on labelled cell concentrations. Statistical significance was determined using a two-sided Mann-Whitney U test comparing each concentration against the 0% control. Brackets indicate significant differences, defined as * p < 0.05, ** p < 0.01, and **** p < 0.0001. Comparisons with non-significant differences are not marked. n > 800

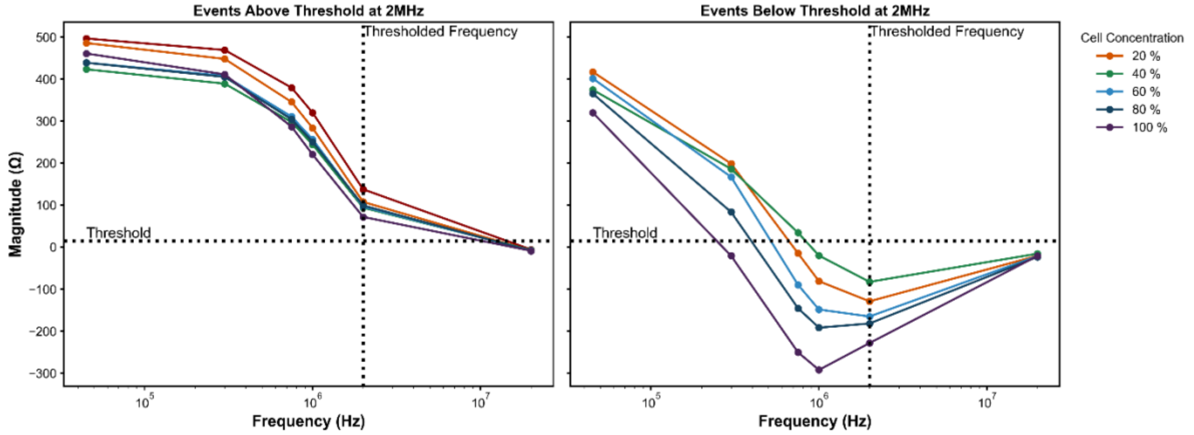

Figure S4: Spectral analysis of events above and below the threshold at 2MHz. Comparison of impedance responses across six discrete frequencies. Data was segregated using the threshold to distinguish high-magnitude events (Left) from low-magnitude events (Right). Data points represent the median value for each concentration.

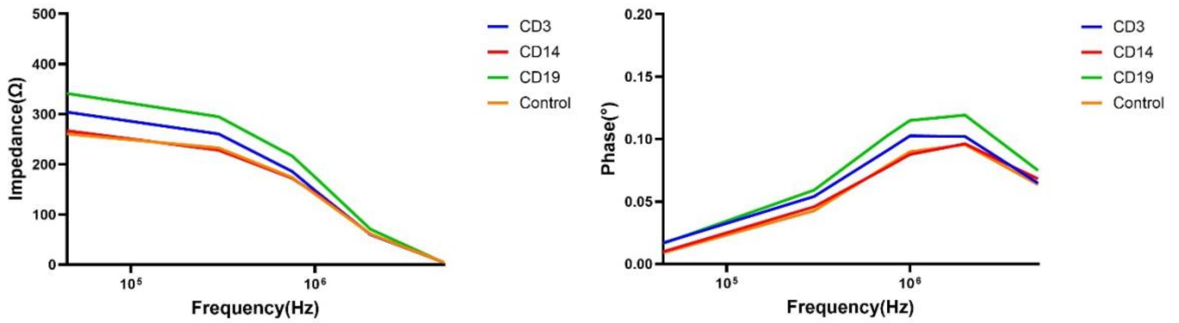

Figure S5: Impedance magnitude and phase change spectra of CD3, CD14, CD19 and control of PBMCs

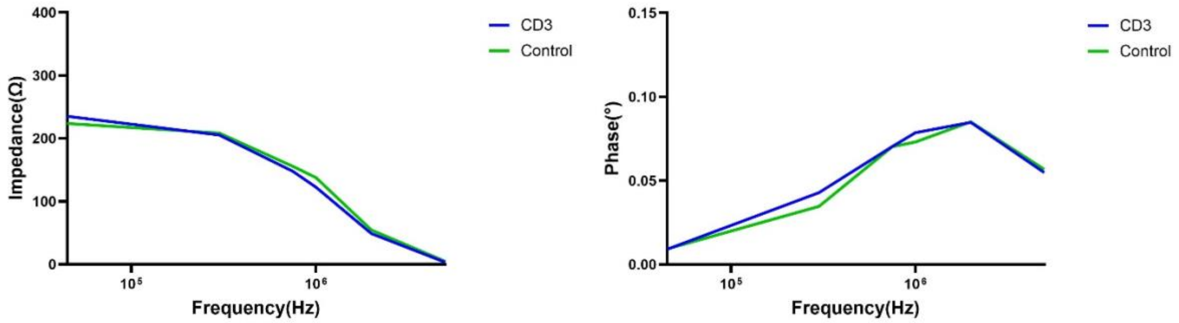

Figure S6: Impedance magnitude and phase change spectra of CD3 and control of T Cells

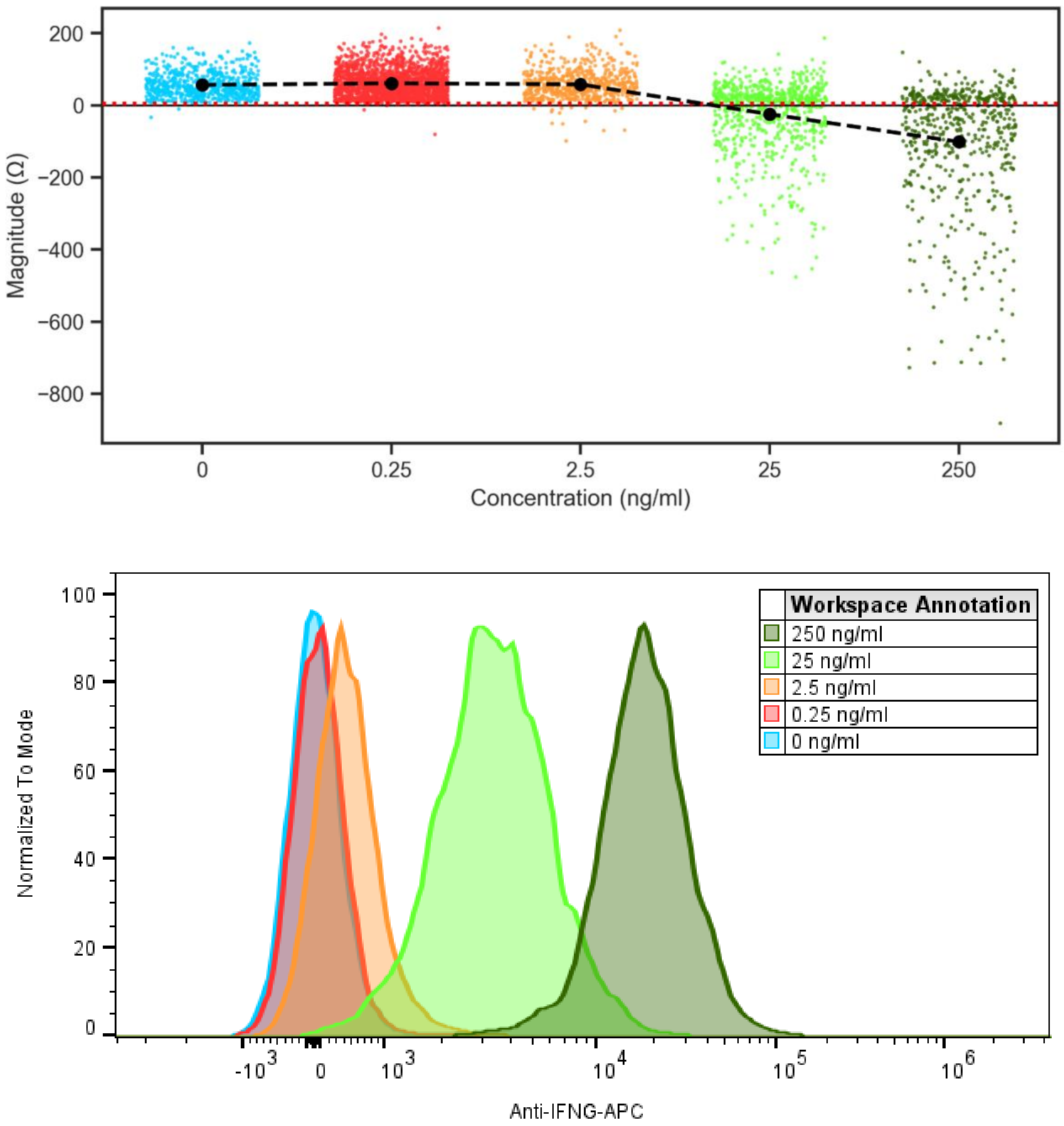

Figure S7: Dilution curve of IFN-G Capture assay of Jurkats dosed with varying concentrations of recombinant IFN-G. Impedance magnitude at 2MHz is shown (Top, n=800). Flow cytometry plot of the same sample (Bottom) showcasing comparable performance of EPIC. Each value represents the concentration added to 10^6 Jurkat Cells.

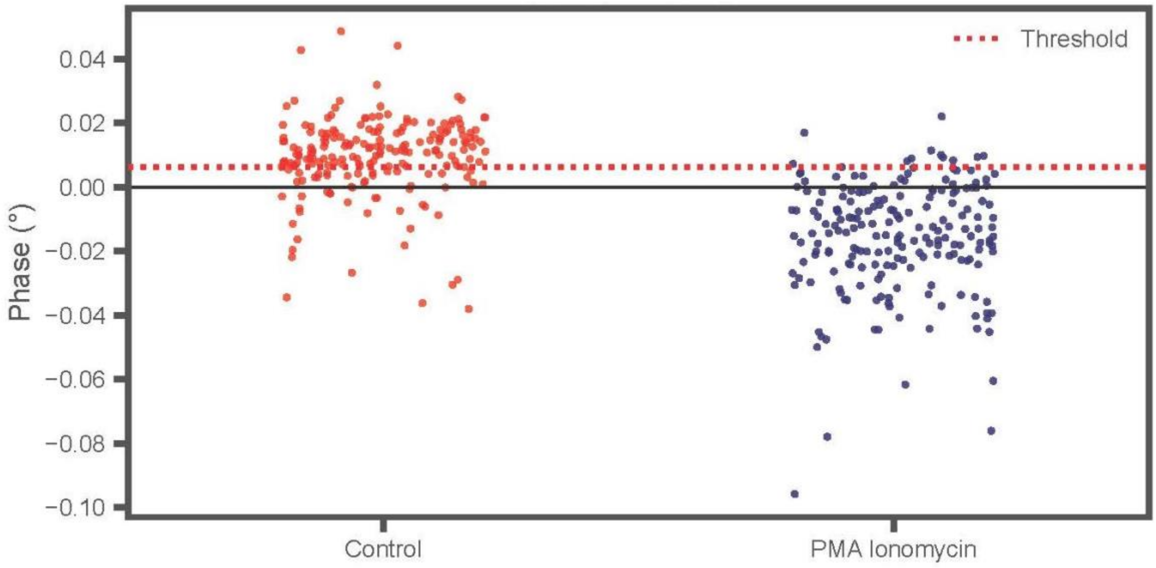

Figure S8: Change in 20MHz Phase between stimulated and control cells

Table S1: Quantitative benchmarking of EPIC versus established clinical assays for functional immune monitoring.

**Feature / Parameter**	**EPIC (This Work)**	**Clinical Flow Cytometry (ICS)[1, 2]**	**Clinical ELISpot (T-SPOT.TB)[3-5]**
**I. System Overview**			
**Primary Readout**	Impedance (Electrical)	Fluorescence (Optical)	Spot Counting (Optical)
**Single-Cell Resolution**	Yes	Yes	Yes (Discrete Spots)
**Portability**	Yes	No	No
**Instrumentation Cost**	<$5,000(Custom electronics) upto $20,000(With lock-in amplifier)	High ($100,000 – $500,000)	Moderate ($25,000 – $75,000)[6]
**Operational Setting**	Decentralized / Point-of-Care	Centralized Core Laboratory	Specialized Clinical Laboratory
**II. Performance Metrics**			
**Min. Detectable Fraction**	0.5% (1 in 200 cells)	~0.02% – 0.05% (Limit of noise)	~0.003% (1 in ~30,000)
**Throughput**	Upto 10,000 cells/min	>10,000 cells/min	Cell count per plate
**Sample Prep Complexity**	Complex, but easily integrated in microfluidic modules	Complex (Fix, Perm, Wash, Centrifuge)	Moderate to complex (PBMC Isolation, Plating, Staining)
**III. Assay Time Breakdown**			
**1. Antigen Stimulation**	5 Hours	6 – 16 Hours *(Antigen-specific requires overnight)*	16 – 20 Hours
**2. Capture / Processing**	45 Minutes *(Surface Capture)*	~3 – 4 Hours *(Fixation, Permeabilization, Staining)*	~1.5 Hours

**3. Signal Amplification**	45 Minutes *(Enzymatic Metallization)*	N/A *(Direct Fluorophore)*	N/A
**4. Readout / Analysis**	5 Minutes	~ 0.5 – 1 Hour	~15 Minutes *(Automated Plate Reader)*
**Total Assay Duration**	~ 6.5 Hours	~ 10 – 21 Hours	~ 18 – 22 Hours

Table S2: Comparison of the current EPIC platform with previously reported metallization-based diagnostic technologies.

**Device**	**EPIC(This Work)**	**BEAD-EM[7]**	**EasyELISA[8]**	**uMAP[9]**	**Optical TB Dx[10]**
**Immune Diagnostic Type**	Cellular	Humoral	Humoral	Humoral	Humoral
**Detection Method**	Metallization (Impedance)	Metallization (Impedance)	Metallization (Impedance)	Metallization (Optical)	Metallization (Optical)
**Substrate**	Cells	Functionalized Microparticles	Functionalized Glass chips	Functionalized Glass Slides	Functionalized Glass Slides
**Sample**	PBMCs	Patient Serum	Patient Serum	Patient Serum	Patient Serum
**Multiplexing**	N/A	N/A	4-plex	25-plex	7-plex

1. Smith, S.G., et al., *Intracellular Cytokine Staining and Flow Cytometry: Considerations for Application in Clinical Trials of Novel Tuberculosis Vaccines.* PLoS One, 2015. **10**(9): p. e0138042.
2. Yin, Y., A. Mitson-Salazar, and C. Prussin, *Detection of Intracellular Cytokines by Flow Cytometry.* Curr Protoc Immunol, 2015. **110**: p. 6 24 1–6 24 18.
3. Berrong, M., D. Nettere, and G. Ferrari, *Methods for Detection of Antigen-Specific T Cells by Enzyme-Linked Immunospot Assay*, in *Manual of Molecular and Clinical Laboratory Immunology*. 2024. p. 277–283.
4. Slota, M., et al., *ELISpot for measuring human immune responses to vaccines.* Expert Rev Vaccines, 2011. **10**(3): p. 299–306.
5. Meier, T., et al., *Sensitivity of a new commercial enzyme-linked immunospot assay (T SPOT-TB) for diagnosis of tuberculosis in clinical practice.* Eur J Clin Microbiol Infect Dis, 2005. **24**(8): p. 529–36.
6. Neuromics. *QHub ELISpot Reader*. Available from: https://www.neuromics.com/MVSCES100?srsltid=AfmBOorqxErD_35k_WxfAAL7VcAt0GIIQWoJwkkNGlLkxXGCAOh5vcaD.
7. Rudge, J., et al., *Electronic Immunoassay Using Enzymatic Metallization on Microparticles.* ACS Omega, 2023.

8. Rafat, N., et al., *Enhanced Enzymatically Amplified Metallization on Nanostructured Surfaces for Multiplexed Point-of-Care Electrical Detection of COVID-19 Biomarkers.* Small, 2022. **18**(49): p. e2203309.
9. Zhang, H., et al., *Sample-sparing multiplexed antibody Fc biomarker discovery using a reconfigurable integrated microfluidic platform.* Lab Chip, 2025. **25**(12): p. 2828–2838.
10. Ali, S.M., et al., *Microscale Multiplexed Antigen-Specific Antibody Fc Profiling for Point-of-Care Diagnosis of Tuberculosis.* medRxiv, 2025: p. 2025.11.15.25340307.
